# ‘We are nothing without herbs’: a story of herbal remedies use during pregnancy in rural Ghana

**DOI:** 10.1186/s12906-019-2476-x

**Published:** 2019-03-15

**Authors:** Prince Peprah, Williams Agyemang-Duah, Francis Arthur-Holmes, Hayford Isaac Budu, Emmanuel Mawuli Abalo, Reforce Okwei, Julius Nyonyo

**Affiliations:** 1 Department of Natural and Built Environment, Sheffield Hallam University, Sheffield, UK; 2 Department of Planning, Kwame Nkrumah University of Science and Technology, Kumasi, Ghana; 3 Oxford Department of International Development, University of Oxford, Oxford, UK; 4 Department of Nursing, Nkrumah University of Science and Technology, Kumasi, Ghana; 5 School of Geography and the Environment, University of Oxford, South-Parks Road, Oxford, GB OX1 3QY UK; 6 Department of Geography and Rural Development, Kwame Nkrumah University of Science and Technology, Kumasi, Ghana

**Keywords:** Rural Ghana, Birim South District, Traditional medicine, Biologically-based products, Pregnant women, Holistic treatment

## Abstract

**Background:**

Herbal medicine has become the panacea for many rural pregnant women in Ghana despite the modern western antenatal care which has developed in most parts of the country. To our knowledge, previous studies investigating herbal medicine use have primarily reported general attitudes and perceptions of use, overlooking the standpoint of pregnant women and their attitudes, and utilisation of herbal medicine in Ghana. Knowledge of herbal medicine use among rural pregnant women and the potential side effects of many herbs in pregnancy are therefore limited in the country; this qualitative study attempts to address this gap by exploring the perceptions of herbal medicine usage among pregnant women in rural Ghana.

**Methods:**

A sample of 30, conveniently selected pregnant women, were involved in this study from April 11 to June 22, 2017. Data from three different focus group discussions were thematically analysed and presented based on an a posteriori inductive reduction approach.

**Results:**

The main findings were that pregnant women used herbal medicine, most commonly ginger, peppermint, thyme, chamomile, aniseeds, green tea, tealeaf, raspberry, and echinacea leaf consistently throughout the three trimesters of pregnancy. Cultural norms and health beliefs in the form of personal philosophies, desire to manage one’s own health, illness perceptions, and a holistic healing approach were ascribed to the widespread use of herbs.

**Conclusion:**

We recommend public education and awareness on disclosure of herbal medicine use to medical practitioners among pregnant women.

## Background

Pregnancy is a condition associated with tremendous physiological changes resulting in many health challenges, including frequent vomiting, heartburn, nausea, and constipation [1]. These ailments, according to Lisha and Nisha [2], often cause pregnant women to resort to self-medication including the use of herbal medicine. Consequently, the use of herbal medicine has increasingly gained more popularity across the globe with women as the main users of these alternative therapies, [3, 4] specifically during pregnancy [5, 6].

Globally, the incidence of herbal medicine usage among pregnant women ranges between 7 and 96% [7–10]. However, variations exist in the utilisation rate of herbal medicine between developed and emerging economies which is largely attributed to cultural differences. For example, in developed countries like Australia, United Kingdom, Italy, Norway, and the United States, the use of herbal medicine among pregnant women ranges from 10 to 56% [9, 11, 12]. The motivation for the use of herbal medicine has, however, been linked to the desire to treat pregnancy-related health ailments, perceived safety and effectiveness, and long personal experience [11, 13]. Moreover, sense of active participation, independence, and control over health and body, and holistic treatment have been reported to serve as additional motivations to use of herbal medicine in the developed countries [14, 15].

However, in developing countries, particularly sub-Saharan African countries, the prevalence of use of herbs among pregnant women is estimated to range from 30 to 70% indicating a higher prevalence of herbs used in Africa than the Western world [16]. For instance, about 35% of pregnant women in Cote d’ Ivoire, 31% of pregnant women in Nigeria, 33% of pregnant women in South Africa and 42% of pregnant women in Tanzania use herbal medicine [8]. This relatively high prevalence is attributed to three main factors: 1) lack of flexible legislation regulating the distribution and purchase of herbal medicine; 2) cultural and personal beliefs and; 3) the high cost of, and low accessibility to conventional medicine and health care [17].

In Ghana, there are scant data on the use of herbs among pregnant women, however, it is openly known that herbal medicine since the precolonial era has played a significant role during pregnancy, delivery and postpartum care in many parts of the country. Especially in rural areas, the demand for herbal medicine by pregnant women has increased over the years. Herbal medicine has, therefore, become a panacea for many rural pregnant women in Ghana despite the modern western antenatal care which has developed in most parts of the country.

Previous studies investigating herb use in Ghana have reported general attitudes, perceptions, and prevalence of use, omitting the standpoint of pregnant women in relation to their attitudes, perceptions and utilisation of herbal medicine. Though such studies exist, they are mostly concentrated in the developed world and are unlikely to be applicable to the rural Ghanaian context lagely due to socio-cultural differences. Hence, this study was conducted with the overarching aim of exploring and bridging the knowledge gap held by rural Ghanaian pregnant women regarding their attitudinal and perceptional factors influencing their utilisation of herbal medicine in Birim South District. Findings from this study are also envisaged to offer useful information to inform policymakers in the health sector of Ghana towards a possible integrative healthcare policy.

### Theoretical approach

There are a number of perspectives that are commonly used to frame research on attitudes, perceptions, and motivation for herbal medicine utilisation. We adopted Lauver’s [18] theory of care-seeking behaviour [CSB] to explore perceptions and attitudes toward herbal medicine among pregnant women in rural Ghana.

**Fig. 1:**
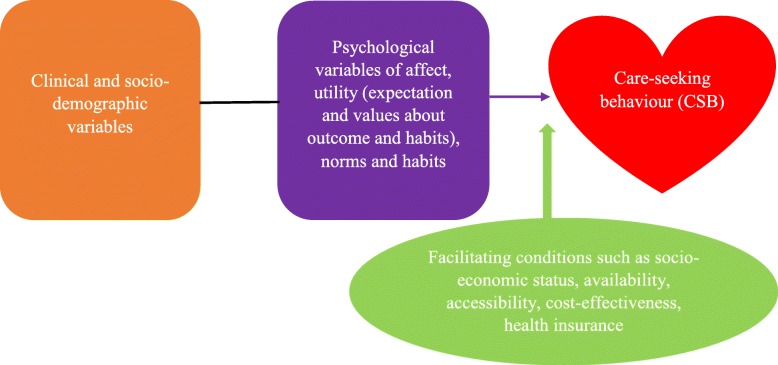
Visual presentation of the CSB process

This section explains the key components (influential factors of care-seeking) of the theory and their interrelationships (Fig. 1). The theory of CSB emerged from Triandis’ [19] general behaviour theory. This theory has been extensively applied in many fields, especially around behavioural changes towards healthcare utilisation among a specific group of people. The probability to engage in certain health behaviour is predominantly influenced by two main variables: psychological variables of affect, utility (expectation and values about outcome), norms and habits; and facilitating variables such as health insurance, accessibility and cost-effectiveness (Fig. 1). In explaining individual variables in the theory as captured in Fig. 1, 'affect' refers to feelings attached to care-seeking, including treatment concerns, and these affectual concerns may have the potential to influence people away from using certain healthcare services. In the context of herbal medicine, people remain committed to the use of herbal medicine and mostly exhibit unwelcome attitudes to the modern healthcare system, which is often considered as culturally-insensitive [20]. Utility refers to the expectation, perceived value and overall care-seeking benefits of individuals. With this, several studies have reported that the perceived efficacy and minimal side effects of herbal medicine are the benefits pulling people to use such therapies [20–22]. According to the theory, norms describe the social, personal and interpersonal issues to capture in care-seeking. Lauver [18] explains that an individual’s own beliefs and personal health philosophies on what is a morally-correct behaviour of seeking care and self-agreement to act based on beliefs by others influence the motivation to access healthcare. Recommendations from family members’, friends’ and relatives’ as well as mass media advertisements influence healthcare decision-making [20, 22]. Moreover, habits represent the way patients act, regarding decision to seek care expeditiously or not. Gyasi et al. [21] emphasise that this can relate more to the care behaviours of previous experiences, especially when a similar situation emerges. However, according to the explanations of the theory, enabling factors such as socio-economic status, affordable medical costs, and health insurance could influence these mechanisms. The facilitating conditions are context specific, objective and external factors that enable one to seek healthcare- thus, they are opposite of conditions that serve as barriers to care-seeking.

As seen in Fig. 1, the relationships among the theoretically identified variables are that psychosocial variables could influence behaviour in interaction with facilitating conditions [19]. The linkages among the variables identified theoretically suggest that psychosocial variables could influence the direction and magnitude of health behaviour either directly or indirectly, and this tendency cannot be underrated. Specifically, the influences of psychosocial variables on behaviour could be direct, whereas variables extrinsic to the theory, such as clinical and demographic factors, are proposed to influence behaviour only indirectly, that is, by mediation through the theoretically identified variables. The study is, therefore, guided by this systematic and inclusive framework that provides insight into how individual health beliefs influence their care decisions. As a theory, the methods and analysis are largely interconnected. The key components (variables influencing care-seeking behaviour) of the CSB theory underpinned the overall methods of the study, especially data collection and analysis techniques. In addition, the discussion section of the study was also guided by the theory as interpretation of the findings was repeatedly linked to specific theoretically identified variables or factors in the theory for meaningful conclusions and implications to be made.

## Methods

### Study design and context

This study adopted a qualitative research approach which allowed the original feelings, experiences and belief systems of participants to be of much importance [23, 24]. These perspectives ensure maximum interaction between the researchers and the interviewees to generate a meaningful collaborative effect [25]. This research orientation was useful because it helped to avoid rigid structural paradigms such as those in positivist research and adopt more personal/flexible research structures which are receptive to capturing meanings in human interaction and making sense of what is perceived as reality. With this, the interviewer and his/her informants are interdependent and mutually interactive and remain open to new knowledge throughout the study, developed with the help of informants [24, 25].

The study focused on the Eastern Region which is popularly known for healthcare and therapeutic pluralism where traditional and conventional medicines are used side by side. However, herbal medicine dominates in this region. This district was chosen also because of its location. The district is located within a semi-deciduous forest landscape, which provides a wide variety of medicinal plant products for traditional and alternative healing purposes. Hence, the district and communities were considered an ideal location for a study that sought to explore pregnant women attitudes, perceptions, and utilisation of herbal medicine.

### The sample and sampling procedure

Participants for the study were a convenient sample of pregnant women in rural communities within the Birim South District who were using herbal medicine prior to the study. The participants were recruited by the researchers themselves from their various homes, clinics/health centres, and workplaces. This was determined by asking pregnant women the question ‘*Are you using herbal medicine*?’ which yielded a ‘yes’ or ‘no’ answer. Here, herbal medicine was defined as a plant’s seeds, berries, roots, leaves, bark, or flowers for medicinal purposes. In all, 50 pregnant women were approached and asked this question, 30 were using herbs, 12 were non-herb users whereas 8 declined to answer the question and subsequently participate in the study. Based on this, 30 pregnant women who were herbal medicine users were included in the study to obtain high-quality information of herbal medicine use among pregnant women in rural Ghana. The sampling technique provided the needed flexibility to focus on participants who were required for the study.

### Data generation tool and procedure

We used focus group discussions for data collection because of its flexibility and openness which enhance unstructured dialogue between the participants and facilitators/moderators for eliciting multiple perspectives about the topic under investigation [26–28]. We conducted three different focus group sessions with each group made up of 10 participants for a detailed and rich information on the subject matter as recommended [29, 30]. The discussions were conducted in *‘Twi’ (the predominant language in the study area)* at open places devoid of third-party interference. Each discussion session lasted for approximately two hours. All participants were assigned numbers for identification. The opening discussion question asked participants to provide details of their experience with regard to specific herbal medicine use. This question generated further arguments and discussions which yielded in-depth data for the study. The discussions were moderated by the researchers themselves to ensure that similar themes and questions were covered in each discussion. All discussions were audio-recorded with the consent of the participants. The moderators made use of cues and prompt to guide and direct the participants into the research topic area; hence they were able to gather more detailed data set. The focus group method has a key limitation pertaining to a propensity for groupthink in that members pressure others to conform to group consensus [31]. Due to the skills of the moderators, conscious monitoring was undertaken to minimise this tendency.

### Data analysis

Data analysis was conducted immediately after all the data were collected to prevent data loss. The analysis involved several steps based on a common set of principles: transcribing the interviews; listening to audiotapes, studying the field or reading the transcripts; developing a data coding system and linking codes or units of data to form overarching categories or themes [32]. To ensure adequate data management, audio records were transcribed and listened, and the responses were typed from the “Twi” dialect into the English language by all the authors individually and cross-checked with the audio records and handwritten field notes to ensure validity, reliability and quality control. The thematic approach of analysis chose by the study is dynamic: it is open to change, driven by the original accounts and observations of the participants and has previously proven to be reliable in a healthcare setting [33, 34]. This technique also allowed researchers to derive themes from the experiences the interviewer obtained from the interactions with the respondents, rather than prior theoretical standpoint of the researchers through a posteriori inductive reduction approach.

Subsequent themes were compared with the responses to identify common trends, similarities, and contrasts. We conducted full data verification where all the transcribed and coded data were checked through proofreading against the original audios and documents to enhance accurate and quality data for the study. The study results were presented under specific broad themes and key subjective views of the participants were presented using quotations.

## Results

The findings of the study constitute the analysis of accounts of the sample recruited for the study. The pregnant women’s positive attitudes, perceptions and regular use of herbs were identified as a core theme and eight interlinking sub-themes were identified to explain the core theme. These were:

- Herbs as the first port of call during pregnancy
- Indigenous knowledge, friends, relatives and the mass media as sources of herbal medicine information
- Personal philosophies and illness perceptions
- Empowerment, control, and participation
- Previous unpleasant experiences of using conventional medicine
- Whole person treatment and perceived efficacy and safety/natural healing
- Perceived good health status because of herbs use
- Poor disclosure habit

### Background information of the study participants

In all, 30 pregnant women participated in the study. Most of our participants were aged between 25 and 35 years (19), currently married (23), Christians (25) and attained only basic school-level education (22). Most participants were self-employed (21) and were dealing in informal economic activities such as traditional peasant farming, artisanal works and petty trading which was reflected in the relatively low-income levels, with the majority receiving monthly income less than GH¢250 ($56.95). Interestingly, most participants (24) had health insurance, which covers medicine at all public and some private healthcare facilities but were still using herbs, providing initial indication for their strong preference for herbal medicine. Table 1 presents the detailed characteristics of the study participants.

**Table 1:** Sample Characteristics

Variables	Categories	N (30)
Age (years)	18–24	7
	25–35	19
	36–45	4
Education	None	3
	Basic	22
	Secondary	3
	Vocational	2
Marital status	Single	2
	Married	23
	Divorced	3
	Widow	2
Employment status	Institutionally employed	2
	Self-employed	21
	Unemployed	7
Household size	1–3	2
	4–6	17
	> 6	11
Health insurance status	Yes	24
	No	6
Average monthly income (GH¢)	< 250	15
	250–350	9
	> 350	6
Religious affiliation	Christianity	25
	Islam	5
Ethnicity	Akan	20
	Others	10

### Herbs as the first resort during pregnancy

Specifically, herbs remained the first therapy used among all the participants with most pregnant women using it once during pregnancy. All the participants were using herbs on regular basis for curative, preventive and health promotion or management therapies. Herbs are the first port of call before formal health visitation in respondents’ vicinities, especially during emergency situations. Most of the participants believed that herbs are part and parcel of their culture and must be used first before any other medicine, when applicable. Other factors reported included availability, accessibility, and cost-effectiveness:

- **Participant three:** *I use herbs almost every week to prevent diseases. You know what? These herbs are accessible, available and very cheap if you are to buy compared to the conventional medicine.*
- **Participant eleven:** *For me, herbs are the main medicine that I use. I have prepared a concoction in my house that I take every morning, afternoon and evening. So, I take herbs every day, except days that it has finished, even when it gets finished, it does not take me more than a day to prepare another one. These herbs are effective, natural and sensitive to our culture, unlike the conventional drugs.*

Interestingly, the study participants showed some specific herbs during the interactions. Participants reported that the most commonly herbs used include: ginger (*Zingiber officinale*), peppermint (*Mentha × piperita*), thyme (*Thymus Lamiaceae*), sage (*Salvia officinalis*), aniseeds (*Pimpinella anisum*), fenugreek (*Trigonella foenum-graecum*), green tea (*Camellia sinensis*), garlic (*Allium sativum*), tea leaf (*Camellia sinensis*), raspberry (*Rubus idaeus****),*** and echinacea leaf (*Echinacea purpurea*). The herbs participants were using were mostly of European origin. The herbs displayed by the participants were for the treatment and prevention of pregnancy-related complications such as relief of back pain, dizziness, stress, and depression, cold, fever, malaria, vomiting, and nausea reduction, as well as to prevent miscarriages. For instance, participants specifically mentioned that ginger and aniseeds are effective for treating many forms of nausea, that sage, and echinacea leaf provide natural treatment to relieve or cure depression, and that peppermint and garlic are effective against the common cold. A combination of boiled tealeaf and fenugreek herbs were described as effective for dizziness, fever, and malaria.

### Indigenous knowledge, friends, relatives and the media as sources of herbal medicine information

Pregnant women have in-depth knowledge about some medicinal plants or herbs that exist in their communities. It became evident that various kinds of medicinal herbs, particularly ginger (*Zingiber officinale*), peppermint (*Mentha × piperita*), tea leaf (*Camellia sinensis*), and raspberry (*Rubus idaeus****)*** have been with them since time immemorial and as a result, knowledge is transmitted from generation to generation and does not require formal education or training for them to know more about medicinal herbs. It emerged from the interviews that ginger, peppermint, and tea leaf are mostly grown or collected at the area or backyard of participants.

**Participant one:** *You see, most medicinal herbs we have in this community dated back in history even pre-colonial period. Our ancestors used them and passed them to us. So to me, knowledge about herbs is transmitted from one generation to another. Personally, I do not think I need formal education to get knowledge about herbal medicines. The same applies to many people in this community, once you are born into herbs usage, you will automatically have knowledge about them.*

Aside from the indigenous knowledge, participants also receive supplementary information about herbs from friends, relatives, family members through recommendations and the mass media (radio and television) via advertisement modules. They specifically mentioned that herbs such as sage, thyme, aniseeds, and fenugreek are mostly bought commercially based on advertisements.

Some shared opinions are presented below:

- **Participant four:** *Though we have some local knowledge about herbal medicine, we also get more information constantly from the mass media. They always advertise and discuss these therapies on televisions and radios which often provide us with information. Mostly, our family members and friends also give us information about some type of herb they know that maybe we are not aware. Have you seen this medicine? I heard about it on the radio that it is very effective for pain relief and I have bought it.*

### Personal philosophies and illness perception

Most of the women's accounts indicated that individual philosophies are directly connected to their unique religious values and the belief systems which influence their healthcare seeking behaviours. The notion that herbs are culturally-sensitive was echoed by the participants with most of them arguing that traditional herbal medicines harmonise with their religious, cultural and spiritual beliefs:

- **Participant five:** *One thing is that herbs have been with us for a very long period and have become part of our tradition, culture, and beliefs. I can tell you with a command that I have great knowledge about these herbal medicines. I know the inside and out of most traditional herbs, we were born into their usage. I can tell you that any member in this community knows specific traditional herbs that he or she relies on. For herbs knowledge, we have it. This is because it is part of our culture unlike the conventional medicine that is alien to us. At least, we should have some form of knowledge about the therapy before using it. But here is the case that we do not have any knowledge about most of the conventional medicine. So, we use herbs because we know how it is and how it works.*

One pregnant woman also related that by explaining how traditional beliefs have influenced her to use herbs throughout her pregnancy period:

- **Participant seven:** *I have a strong belief in the potency of herbs. I know that herbs are part of our culture and total upbringing. I was born into herbs use and have grown in it. It always yields good results when I use it to treat any disease I may suffer from. I think if not herbs, I would have died a long time ago. Herb is my saviour.*

It was further observed that most of the participants believed certain diseases in pregnancy and complications during childbirth have strong spiritual connections of which conventional medicine cannot cure. Pregnant women strongly argued that using herbs from the onset of pregnancy period helps prevent these unforeseen spiritually-motivated diseases. Thus, participants perceived spiritual illness as a reason to use certain herbs:

- ** Participant twenty-five:** *Just like most pregnant women in this community, I started using herbs right after pregnancy was confirmed. I do this because, without such herbs, the likelihood that my child would be affected with diseases is very high. So, I use it to prevent both physical and spiritual diseases which can affect my pregnancy.*

Another participant also related with much emphasis on spirituality:

- **Participant nineteen:** *You see …*. *When you are pregnant several eyes are watching you and not all these eyes are good, some of the eyes watch you with bad intentions to destroy you the parent or the unborn child. To prevent this, we use certain herbs that can prevent such unforeseen diseases with spiritual motivations. Because spiritual diseases must be tackled spiritually through the application of spiritual herbs.*

### Empowerment, control and participation

The desire to take full responsibility and manage their own health and healing during and after childbirth was observed to be another factor that influenced participants to use herbs. In addition, the women emphasised that the use of herbs empower as well as enhancing their confidence to birth their babies in their own abilities:

- ** Participant twenty-four:** *I cannot allow someone to control my health for me. This is because I know myself more than the person [Medical doctor or trained health professional]. I know what is good for me in terms of medicine. I have to control my own health.*

They interestingly explained that allowing someone to take full responsibility and control of their health may not lead to good health outcomes:

- **Participant twelve:** *What is the guarantee that I can give birth with no complication if I do not use herbs? You know what … these herbs help us to be confident that we can give birth naturally without the help of any midwife. These herbs have helped me birthed four children with no complication at any point in my deliveries. So, I will continue to use them to empower me to take control and responsibility for my health.*

### Previous unpleasant experiences of using conventional medicine

Most of the participants also ascribed dissatisfaction and prior unpleasant experiences with the use of conventional medicine as reasons for using herbs. Previous unfortunate experiences, particularly perceived ineffectiveness and the adverse side effects of conventional medicine have influenced participants’ attitudes and perceptions toward conventional medicine and thereby pushed  them towards herbs use:

- **Participant six:** *Conventional medicine is mostly ineffective, chemically-infested, expensive and has serious health implications, at the end, these conventional medicines will just cure signs and symptoms and not the disease itself. I remember I was suffering from malaria as the doctor confirmed. I was given several medicines of which I took them all but the malaria did not go. I battled this for more than two months. Someone recommended one herbal mixture to me and I took it for only three days to feel better. Do you think with this experience I will still use conventional medicine?*

In relation to the above comment, one participant also noted that:

- **Participant eleven:** *I will always use herbal medicine due to what I have observed about most of the conventional medicine I have used previously. I mostly vomit and feel dizzy whenever I take those conventional medicines. So, I am always afraid to take it.*

### Whole person treatment and perceived efficacy, safety and natural healing

The study participants viewed the application of herbs as a holistic approach to healing where whole person treatment is conducted by viewing health and disease through the integration of mind, body, and spirit. Participants clearly elucidated that herbs do not mostly target one particular disease but cure holistically with no or minimal effects:

- **Participant five:** *Herb is everything to me and I really respect these therapies. I see herbs as effective medicine that can heal all manner of diseases with no or minimal side effects even when one takes it overdose. To me, I am nothing without herbs.*
- **Participant thirty:** *I am speaking for myself and other pregnant women in this community, without herbs, most pregnant women would have been dead long ago due to the fact that most conventional medicine given to us by healthcare providers are ineffective. It is capable of treating as many as diseases at the same time. So, we have a special respect for herbs.*

In addition, most of the participants recognised the spiritual dimension of childbirth, as childbirth is a healing, life-changing event and herbs were believed to be effective for tackling perceived spiritual diseases:

- **Participant twenty:** *You will agree with me that midwives at the health centre only deal with diseases in the physical realm. However, most pregnancy-related illnesses is spiritually-motivated which need to be cured and prevented through certain traditional herbs and concoction known to be effective for such illness.*

The safety of conventional therapies was a subject of major concern for the study participants. Most respondents mentioned that prescribed medicine contain chemicals that may have both momentary and long-term side effects on their pregnancy. On the contrary, they perceived herbs to be safe and devoid of adverse side effects. It was further observed that the belief that herbs are safe was based on their understanding of the notion of “natural being neutral”. Thus, herbs appear natural and therefore considered to be safe for use:

- **Participant thirty:** *Natural plants are mostly free from health-threatening chemicals unlike manufactured medicine from a hospital or a chemist’s shop. They (herbal medicine) are safe because they are natural.*

### Perceived good health status as a result of herbs use

Pregnant women perceived their health status during the study to be very good and attributed it to the regular use and perceived effectiveness of herbs:

- **Participant ten:** *I see my health status as very good, I hardly fall sick and I believe is because of the traditional herbal medicine that I regularly use.*

Interestingly, our study participants shifted the discussion by comparing their health status to other pregnant women who they know to be not herb users in the community. The participants perceived their health status as better than the status of pregnant women who use conventional medicine. They explained that pregnant women who use conventional medicine mostly report of sickness and often visit hospitals for health care, meanwhile users of herbs hardly report sickness:

- **Participant twenty-two:** *I can say my health status is very good. I must attest to the fact that my health status was not good when I was using orthodox medicine for disease treatment. With herbs recommended by a friend, my health status has been very good. Moreover, what I have observed in this community for a long time is that I see that pregnant women who rely solely on conventional medicine and health care mostly report illness than the herb users. In all, I see the herb users to be healthier than the conventional medicine users in this community.*

### Poor disclosure habit

The study found that pregnant women occasionally used these herbs alongside with the conventional drugs, but they rarely disclose this to health professionals during hospital visits. Principal reasons highlighted by pregnant women to account for this non-disclosure included beliefs that herbs are natural and safe, fear of losing control over their health decisions and fear that health professionals would victimise, reproach, discourage and possibly stop them from using herbs:

- **Participant twenty:** *Most at times we do not tell the doctors and midwives about our use of herbs, even when they ask us we replied no. To me, when you tell them, they might not be interested and as a result, stop you from using these therapies. Even, those medical professionals who are strongly against these herbs can reproach and discourage you from using herbs.*

Also, some participants saw no benefit in disclosing herb use to professionals who they think did not have adequate knowledge about these herbs and are mostly alien to their traditional settings including medicine:

- **Participant sixteen:** *Personally, I see no benefit in the disclosure since most health professionals do not know much about these herbs and are also alien to our culture. The doctors have asked me several times, but I have never replied yes, meanwhile I use it and I will continue to use it.*

## Discussion

This study has detailed a spectrum of attitudes, perception, and utilisation of herbal medicine among pregnant women in rural Ghana. Empirical studies exploring socio-demographic characteristics of pregnant women who use herbs have reported divergent results due to variations in geographical and economic settings, particularly between developed and developing countries. In addition, cultural settings and access to conventional medicine and healthcare services may have an important role in these variations [35]. In a manner inconsistent with the findings of previous studies on herbal medicine user characteristics [4, 36, 37, 38], this study revealed that pregnant women using herbal medicine mostly had low incomes as well as low levels of education [39]. In consonance with the facilitating variables of accessibility and cost-effectiveness in Fig. 1, the pregnant women studied are enabled to use herbs particularly due to limited income and accessibility. This is because in most rural areas in Ghana, local herbs are mostly free and easy to be found usually at the backyard of residences.

The study found that pregnant women have in-depth knowledge about some herbal medicines which are common to them. As previous research has found [35], knowledge about herbs is transferred from one generation to the other with users capable of correctly classifying, describing and identifying the herbal products they used. Meanwhile, information on herbs which are of foreign origin is becoming accessible through advertisements on various media outlets such as television and radio.

In line with the explanations of the CSB theory, the study found that personal philosophies, attitudes, trust, and satisfaction are critical factors that mostly dictate the use of a healing modality. It can be seen from the explanation of Fig. 1 that factors influencing participants’ use of herbal medicines are related to both facilitating conditions (satisfaction and trust) and norms (personal philosophies and attitudes). Thus, in relation to Fig. 1, participants were enabled to use herbal medicine due to their levels of satisfaction, trust in the herbs, and personal beliefs about what is morally correct behaviour. For instance, regarding the element of norms in Fig. 1, in most rural areas in Ghana, pregnancy events are interpreted with spiritual dimensions and specific complications such as delay in the delivering process and miscarriages are attributed to witchcraft and gods; pregnant women are thus often motivated from on the onset of pregnancy period to use certain herbal medicine that they believe to have proved effective in preventing such unforeseen events.

Meanwhile, patients’ attitudes, trust levels, and satisfaction are important indicators of quality of healthcare and play a very critical role in influencing patients’ choice of health care providers [40–43]. Similarly, Pascoe explained that patients’ satisfaction information can provide a dependent measure of service quality and serves as a predictor of the health-related behaviour of patients [44]. Patient satisfaction may be measured in terms of satisfaction with medical care, satisfaction with providers, and satisfaction with outcomes of treatment [45]. The trust and satisfaction gained by pregnant women with the use of herbal medicine have influenced their perceptions and attitudes towards herbal medicine. These attitudes and perceptions of pregnant women toward herbs reflect their personal experiences as well as their levels of individual exposure to herbs in the past and present. People may have positive perceptions and attitudes towards herbal medicine due to their beliefs which are congruent with traditional healing practices.

Copious contemporary studies in both developed and developing settings, urban and rural settings have ascertained frequent and high uptake of herbal medicine among pregnant women for diverse reasons [46–49]. Similarly, this study demonstrated frequent use of herbal medicine among pregnant women in rural Ghana. The most frequently used herbal medicine found among pregnant women were ginger (*Zingiber officinale*), peppermint (*Mentha × piperita*), thyme (*Thymus Lamiaceae*), sage (*Salvia officinalis*), aniseeds (*Pimpinella anisum*), fenugreek (*Trigonella foenum-graecum*), green tea (*Camellia sinensis*), garlic (*Allium sativum*), tea leaf (*Camellia sinensis*), raspberry (*Rubus idaeus****), and*** echinacea leaf (*Echinacea purpurea*). These herbs used by pregnant women in rural Ghana as found by the study have been reported by previous studies in both developed and developing countries [2, 35, 46, 50–57]. Similar to other study findings [46, 53, 58, 59], these herbs were first used on daily basis before any other medicine with most pregnant women using them consistently throughout the three trimesters of pregnancy. However, what makes this finding extremely interesting is that most of these herbs used by the sampled  pregnant women in rural Ghana were mostly of European and Asian origin, not traditional herbal medicines originating from Ghana. This result can be attributed to mass media campaigns, migration, and colonial influence. The establishment of media outlets in a form of diverse radio waves and telecasts have emerged and contributed immensely to the widespread information about herbs through constant announcements and advertising modules. These media outlets together with recommendations by relatives and friends served as significant knowledge sources and awareness proxies for herbal medicines [2, 35, 40]. This finding implies that more pregnant women are likely to continue using these herbs since information and awareness of herbal medicines are more likely to spread across the population in the Region considering the increasing spread of these media outlets and its associated herbal medicine information dissemination in Ghana.

Various reasons accounting for this regular use of herbal medicine among pregnant women were associated with cultural, social and economic undertones, which are inconsistent with previous studies [6, 8, 15, 38]. The perceived effectiveness, safety, cultural sensitivity, holism, and desire to have control over their health during and after childbirth were the main mediating factors influencing pregnant women’s attitudes, perceptions and the resultant use of herbs. This can also be linked to the utility variable (expectations and values about outcomes) in Fig. 1. Participants were mostly using herbs with the idea that herbs are effective for pregnancy-related ailments such as back pain, dizziness, stress, cold, fever, malaria, vomiting, and nausea, which reflects the overall benefits of using herbs.

The argument by pregnant women using herbs was that conventional medicine is not very effective in dealing with most pregnancy-related diseases. Second, the respondents bemoaned the fact that upon the use of these medications, the numerous adverse reactions in the form of side effects threatened the safety of the patients w. In comparison, however, even the perceived  safety and effectiveness associated with herbs has been keenly contested since there is no substantial empirical evidence and verifiable data explaining the safety of most herbs used by pregnant women, especially those in rural areas. For instance, Nordeng et al. [38] iterated that safety, is held to be an important feature of any treatments and interventions by many pregnant women often resulting in attempts to avoid pharmaceutical treatments during pregnancy or to approach the use of such therapeutic options with caution [56]. Most pregnant women in our study and other studies elsewhere often explain the naturality of herbs to mean safety and do not envisage any side effects of these herbs' consumption during pregnancy [15]. The perceived effectiveness of herbs is more important to pregnant women than the possible side effects [60]. This finding suggests that, although pregnant women exhibited positive perception and attitudes towards herbs as well as frequent use of herbal medicines, there is an urgent need to validate the quality of these traditional herbal medicines through randomised clinical trials and test to prove these herbs safety in rural Ghana where herbal medicine is widespread; less expensive, more readily available, accessible and closer to the people than conventional ones.

One area that has generated tremendous debate in midwifery recently is the belief of midwives and obstetricians supremacy over childbirth [61, 62]. There is a belief that midwives deny pregnant women the right to have some sort of control over their own health issues and body as a whole [61, 62]. In addition, supporters of biomedicine have argued that scientific medical practices are purely concerned with disease and science as well as principles and methods to the neglect of environmental and social issues in favour of biological ones [63, 64]. This scientific medical practice is often argued to treat diseases, rather than the person and mostly overlook patient’s individual experience [65, 66]. With scientific medical approach, health is seen as holistic while herbal medicine use provides a unique holistic way to treat not just an aspect of the being and or disease-specific but a whole being, considering the importance of body, mind, and spirit. The notion that herbal medicine conveniently deals with physical, spiritual and or emotional problems towards a “whole health” restoration by pregnant women pushes them to take herbs regularly [67–69]. This implies that holism remains one major concept that separates the traditional system of medicine and the conventional counterpart. These sentiments shared by pregnant women who formed the main participants and unit of analysis in the study reflect similar feelings described in earlier studies which found that pregnant valued self-confidence in their capacity to manage their own health issues, linked to sentiments of autonomy and control [70, 71]. This concept of a whole person approach to health and well-being may be useful as advocated by World Health Organisation [WHO] that health is “a state of complete physical, mental and social well-being and not merely absent of disease or infirmity”.

Ben-Ayre and Frenkel [72] argued that the popularity of complementary and alternative medicine [CAM] therapies of which herbal medicine is part, has affected doctor-patient communication as patients do not disclose their CAM use to medical practitioners. In fact, this study reveals that herbal medicines were often used by pregnant women without the knowledge and support of medical practitioners. The herbs used were mostly self-prepared by combining different kinds and parts of plants. In agreement with other empirical findings [8, 35, 48], pregnant women intentionally declined to disclose their use of herbs to their health professionals, even when asked, but preferred to seek advice from family and friends. The non-disclosure habit was found to be associated with the belief that herbs are natural and safe, that health professionals would create feelings of victimisation, reproach, discouragement and possibly stop their use of herbal medicine, the fear of relinquishing control over decisions concerning their wellbeing and health, and the belief that health professionals do not have adequate knowledge concerning the herbs used [15, 46]. In contradiction, Tsui et al. [73] found that 70% of their Californian respondents had reported their herbal medicine use to a primary healthcare practitioner or a doctor. This inconsistency could be because of difference in the study settings and methodological approaches.

The results of this study identified good perceived health status among pregnant women who were herb users. Pregnant women rated their health status as good and attributed it to the regular use of herbs. However, these women emphatically mentioned that their health status was far better than their non-users' counterpart when comparing their rates of sickness complaints and number of hospital visits. Pregnant women who used herbs argued that non-users of herbal medicine frequently complain of ailments and as a result visit hospitals on regular basis. With this, our study participants maintained that using herbal medicines could relieve and prevent diseases and thus; promote good health during and after pregnancy.

It is also important to note that the CSB theory that guided the study cannot completely explain the findings of the study. For instance, the perceived health status of participants cannot be explained by the theory. This is mainly because the theory discusses behaviours toward care-seeking, and does not explain what the health status of individuals would be after engaging in a particular health behaviour. This probably calls for an extension of the theory to include health status.

Some strengths and limitations of this study need to be acknowledged to inform readers to put the interpretation of the study findings into its right context and perspective. To best of our knowledge, this is the first study to provide insights into the rural pregnant women’s perspective of perceptions, attitudes, and utilisation of herbal medicine in Ghana. The study, therefore, demonstrates a depth of understanding from the views of persons of beneficiaries of herbal medicine use (pregnant women) and offers an important contribution to address the existing gap in knowledge. It also probes Ghana’s health policy framework on a potential regulation of intercultural healthcare system. However, the study has some limitations which are premised on its methods; particularly sample size and sampling procedures. This study consciously prioritised the depth of participants’ experiences, rather than merely the breadth. The authors believe that the limitations are far outmatched by the benefits offered by conducting this first empirical study on herbal medicine perceptions, attitudes, and utilisation among pregnant women in rural Ghana.

## Conclusion

This qualitative study explores the attitudes, perceptions, and utilisation of herbal medicine among pregnant women in rural Ghana. Many pregnant women reported positive perceptions and attitudes towards herbs as well as using herbal medicine therapies frequently. However, rural pregnant women predominantly ascribed perceived efficacy, safety, control and autonomy over health issues, holistic approach of herbs, availability, accessibility, and cost-effectiveness as reasons for the widespread herbal medicine use. Therefore, we recommend public education and awareness on disclosure of herbal medicine use to medical practitioners among pregnant women. Moreover, the study findings call for further clinical research into the safety of most herbs used by pregnant women through appropriate institutions such as the Ghana Health Service.

## Abbreviations

CAM: Complementary and Alternative Medicine
CSB: Care-Seeking Behaviour
WHO: World Health Organisation
